# Diversity among isolates of *Staphylococcus intermedius* group from Pacific marine mammals of Southern California

**DOI:** 10.1371/journal.pone.0356088

**Published:** 2026-08-24

**Authors:** Igor Loncaric, Lauren Palmer, Michael P. Szostak, Adriana Cabal Rosel, Werner Ruppitsch, Markus Rupprecht, Amelie Desvars-Larrive, Olivia M. Grünzweil, Elke Müller, Sascha D. Braun, Stefan Monecke, Ralf Ehricht, Andrea T. Feßler, Stefan Schwarz, Joachim Spergser, Hrvoje Smodlaka, Suzana Tkalcic

**Affiliations:** 1 Microbiology, University of Veterinary Medicine Vienna, Vienna, Austria; 2 Marine Mammal Care Center, Los Angeles, California, United States of America; 3 Division Public Health, Institute for Surveillance & Infectious Disease Epidemiology, Austrian Agency for Health and Food Safety, Vienna, Austria; 4 Institute of Hygiene and Medical Microbiology, Medical University Innsbruck, Innsbruck, Austria; 5 Faculty of Food Technology, Food Safety and Ecology, University of Donja Gorica, Podgorica, Montenegro; 6 Clinical Department for Farm Animals and Food System Transformation, University of Veterinary Medicine Vienna, Vienna, Austria; 7 Complexity Science Hub, Vienna, Austria; 8 Leibniz-Institute of Photonic Technology (Leibniz-IPHT), Jena, Germany, member of the Leibniz Center for Photonics in Infection Research (LPI), Jena, Germany; 9 InfectoGnostics Research Campus, Jena, Germany; 10 Center for Translational Medicine (CETRAMED), Jena University Hospital, Friedrich Schiller University Jena, Jena, Germany; 11 Institute of Physical Chemistry, Friedrich Schiller University Jena, Jena, Germany; 12 Institute of Microbiology and Epizootics, Centre for Infection Medicine, School of Veterinary Medicine, Freie Universität Berlin, Berlin, Germany; 13 Veterinary Centre for Resistance Research (TZR), School of Veterinary Medicine, Freie Universität Berlin, Berlin, Germany; 14 College of Veterinary Medicine, Western University of Health Sciences, Pomona, California, United States of America; University of Calgary, CANADA

## Abstract

This study investigated the carriage of *Staphylococcus* (*S.*) *intermedius* group (SIG) isolates in marine mammals in the Pacific coastal region of Southern California, USA. We screened 452 samples from the oral cavities of California sea lions (*Zalophus californianus*), northern elephant seals (*Mirounga angustirostris*), harbor seals (*Phoca vitulina*), northern fur seals (*Callorhinus ursinus*), Guadalupe fur seals (*Arctocephalus townsendi*), long beaked common dolphin (*Delphinus delphis bairdii*), Pacific white-sided dolphins (*Sagmatias obliquidens*)*,* and pygmy sperm whales (*Kogia breviceps*). Forty-two *S. pseudintermedius* and four *S. delphini* were obtained. Isolates were characterized by a polyphasic approach, which included antimicrobial susceptibility testing, molecular identification and characterization by *rpoB/nuc* sequencing, PCRs, DNA-based microarray, genotyping via *spa* and MLVA, and whole-genome sequencing of selected isolates. Resistance to penicillin, penicillin/tetracycline, and tetracycline alone was observed. *Spa* typing resulted either in novel types or in isolates that were non‑typeable. Twenty-nine MLVA types were detected. Multi-locus sequence typing revealed four STs: ST453 (previously described) and three novel STs: ST3046−8) among *S. pseudintermedius* and various virulence-associated genes were detected. Long-term monitoring remains essential to fully map its epidemiology, pathogenicity, and evolution of SIG in marine mammals.

## Introduction

The North American Pacific coastline is home to a diverse assemblage of marine mammals, encompassing approximately 50 cetacean and16 pinniped [1]. Common pinnipeds in this region include the California sea lion (*Zalophus californianus*), northern fur seal (*Callorhinus ursinus*), harbor seal (*Phoca vitulina*), and northern elephant seal (*Mirounga angustirostris*), as well as threatened species such as the Guadalupe fur seal (*Arctocephalus townsendi*). These animals are recognized sentinels of ocean health, with their population health and disease status reflecting the equilibrium of the marine ecosystems they inhabit [2,3]. In California, primary health threats to marine mammals include human-inflicted trauma, biotoxins from harmful algal blooms, and bacterial infections [4]. Critically, because these species share coastal environments and trophic resources with humans, monitoring their health is central to a One Health approach for assessing ocean health and mitigating potential zoonotic disease risks [5].

The *Staphylococcus intermedius* group (SIG) comprises closely related coagulase-positive bacteria, including *S. intermedius*, *Staphylococcus pseudintermedius*, and *Staphylococcus delphini*. More recently, novel species have been included in the group, such as *Staphylococcus cornubiensis*, isolated from a human clinical case [6–9] and *Staphylococcus ursi*, initially isolated from an American black bear [10,11].

SIG members typically colonize the skin and mucous membranes of a broad range of hosts, including companion animals, livestock, and wildlife such as minks, dolphins, pigeons, dogs, horses, and cats [7,12–17] and are involved in the etiology of bacterial otitis, dermatitis, and other soft-tissue infections in domestic animals and wildlife as primary or secondary pathogens [12,15,18]. Although less common, human infections caused by SIG members have also been reported, and some cases historically attributed to *S. aureus* were later reclassified as SIG species [16,17,19]. Recent molecular and genomic studies have refined the taxonomy of this group, revealing significant genetic diversity among SIG members. Many isolates previously identified as *S. intermedius* have been correctly reclassified as *S. pseudintermedius* [12,18,20–22]. Continued investigation of the SIG taxonomy and host associations is essential to improve diagnostic accuracy and to better understand their epidemiological and zoonotic potential.

The Californian coastline includes many densely populated urban areas capable of generating large volumes of contaminated wastewater (1.58 billion m^3^ in 2015 [23]), which deposit into the ocean potentially leading to widespread dispersal of the zoonotic SIG, including antibiotic-resistant variants. Consequently, microorganisms traditionally associated with terrestrial mammals may infect marine mammals, posing potential health risks to these marine species. The aim of this study is to characterize the diversity of *S. intermedius* group isolates circulating in marine mammals along the Pacific coast of Southern California, USA.

## Materials and methods

### Isolation of *Staphylococcus intermedius* group strains

Oral samples were collected from stranded marine mammals from October 2019 to September 2022 upon admission to the Marine Mammal Care Center, Los Angeles (MMCCLA) as part of a diagnostic health evaluation, and, therefore, the approval of the local ethics committee was not required. In total, 451 oral samples from 345 California sea lions (*Z. californianus*), 75 northern elephant seals (*M. angustirostris*), 11 harbor seals (*P. vitulina*), nine northern fur seals (*Callorhinus ursinus*), five Guadalupe fur seals (*Arctocephalus townsendi*), three long beaked common dolphins (*D. delphis bairdii*), two Pacific white-sided dolphins (*S. obliquidens*), and one pygmy sperm whale (*K. breviceps*) (S1 Table). For the isolation of staphylococci each oral swab was incubated at 37°C overnight in tryptic soy broth (BD, Heidelberg, Germany) with 6.5% (w/v) NaCl and subsequently streaked onto BD™ Columbia CNA Agar with 5% Sheep Blood, Improved II (BD, Heidelberg, Germany). Bacterial colonies showing the typical comorphology for members of SIG were further confirmed by matrix-assisted laser desorption ionization-time-of-flight mass spectrometry (MALDI-TOF MS) (Bruker Daltonik, Bremen, Germany) and were cryo-preserved at −80°C for subsequent analyses.

We determined the proportions of SIG-positive per species and estimated 95% confidence intervals (CIs) using the Clopper-Pearson exact method via the binom.test function in R [24].

### Antimicrobial susceptibility testing

Antimicrobial susceptibility testing was performed using agar disk diffusion according to CLSI [25]. The following antimicrobial agents were used: penicillin (10 units), oxacillin (1 μg), ciprofloxacin (5 μg), gentamicin (10 μg), tetracycline (30 μg), erythromycin (15 μg), clindamycin (2 μg), chloramphenicol (30 μg), trimethoprim-sulfamethoxazole (1.25/23.75 μg), nitrofurantoin (300 μg), rifampicin (5 μg), and linezolid (30 μg) (all from Becton Dickinson, Heidelberg, Germany). *S. aureus* ATCC® 25923 served as the quality control strain.

### Molecular characterization of isolates

Genomic DNA of staphylococci was extracted using the Qiagen DNeasy Blood & Tissue Kit (Qiagen GmbH, Hilden, Germany). Prior to extraction, initial lysis was performed using the lysis enhancer and buffer provided with the INTER-ARRAY Genotyping Kit *S. aureus* (Bad Langensalza, Germany). For final species allocation, *rpoB* and RNA polymerase β-*nuc* sequencing were used [26,27]. Phylogenetic relationships among isolates were inferred using the unrooted neighbor-joining method. Sequence alignment, analysis, and visualization were conducted with Molecular Evolutionary Genetics Analysis software version 11 (MEGA 11) [28].

All isolates were subjected to staphylococcal protein A *(spa)* typing following the protocol of Zukancic et al. [29]. PCR products yielding positive bands by gel electrophoresis were Sanger sequenced. *spa* types have been assigned as described previously [30] and based on the offline database provided by Arshnee Moodley. In addition, isolates were genotyped using Multi-Locus Variable Number of Tandem Repeat Analysis (MLVA), which relies on variable numbers of tandem repeats (VNTRs) [31]. To identify loci suitable for MLVA of *S. pseudintermedius*, an *in silico* analysis was performed on reference strain ED99 (GenBank accession CP002478) [32] using Tandem Repeats Finder, (https://tandem.bu.edu/trf/home, last accesses February 2026, [33]. Eight identified loci were selected for this study, with primers and annealing temperatures listed in S2 Table. This selection was based on the following criteria: (i) minimum repeat size of 30 bp, (ii) conservation between the tandem repeats (>90%), and (iii) a copy number ≥2. Primers were designed for the region flanking each VNTR using Primer3 (https://primer3.ut.ee/) [34]. Amplicon sizes were determined by gel electrophoresis. The number of repeats at each locus was calculated using the formula:


$$\begin{array}{c} \;\text{Number of repeats}=\frac{\text{Amplicon size}-\text{Flanking region size}}{\text{Repeat size in ED99}}, \end{array}$$

and rounded up to an even number [35]. This created an eight-digit code of repeats in each locus and was utilized for generating the MLVA types. Genetic relationships were analyzed using the categorical coefficient (Hamming distance), with clustering performed via the Unweighted Pair Group Method with Arithmetic Mean (UPGMA) integrated in PHYLOViZ 2.0 [36]. All isolates were screened with PCR for six virulence-associated genes, including three exfoliative toxin genes (*siet*, *expA*, and *expB*) [37–40], the leucocidin locus *lukI* (comprising *lukS* and *lukF*) [41], and the canine staphylococcal enterotoxin C gene (*secCanine*) [40]. In addition, all isolates were analyzed using the Genotyping Kit *S. aureus* (INTER-ARRAY, fmzb GmbH, Bad Langensalza, Germany) following the manufacturer’s instructions [42,43].

Eight isolates were selected for whole-genome sequencing (WGS), seven *S. pseuditermedius* isolates were selected based on phenotypic resistance, *spa* type, and MLVA type. None of the isolates shared all characteristics. Additionally, one *S. dephini* isolate was selected at random. Isolate DNA extraction, WGS and genome assembly were performed as previously described [44]. Briefly, Genomic DNA was isolated using the MagAttract HMW DNA Kit (Qiagen, Hilden, Germany), and sequencing libraries were prepared with the Nextera XT DNA Library Preparation Kit (Illumina, San Diego, CA, USA). Paired-end sequencing (2 x 150 bp) was performed on an Illumina NextSeq 2000 platform, targeting a minimum coverage depth of 30-fold. Following quality trimming of the raw reads, de novo genome assembly was carried out using SPAdes v3.11.1. The species allocation was verified by JSpecies workspace using the ANIb (average nucleotide identity via BLAST) analysis tool [45]. Sequence types (ST) were assigned using the database hosted at PubMLST (https://pubmlst.org/organisms/staphylococcus-pseudintermedius) [46]. The clonal relationships of the identified STs were assessed by integrating them with entries from the PubMLST *Staphylococcus pseudintermedius* database. All database entries available at the time of analysis (February 2026) were clustered using the goeBURST algorithm implemented in the PHYLOViZ 2.0 [36]. To identify acquired resistance genes and/or chromosomal mutations, ResFinder 4.7.2. with default setting (https://genepi.food.dtu.dk/resfinder/, accessed February 2026) was used [47]. ABRicate [48] v1.2.0 was used to identify antimicrobial resistance genes using Comprehensive Antibiotic Resistance Database (CARD) [49], as well as genes associated with biocide resistance with Antibacterial Biocide and Metal Resistance Genes Database (BacMet) [50]. Virulence-associated genes were identified by using Virulence Factor Database (VFDB, https://www.mgc.ac.cn/VFs/main.htm, last accessed February 2026) [51].The Bacterial and Viral Bioinformatics Resource Center (BV-BRC) [52] was used for gene prediction and annotation. All genomes were uploaded to BV-BRC, and genes were annotated and predicted using the Genome Annotation tool. The DNA sequences of the *siet* and *expB* genes from the reference genome ED99 (CP002478.1) were BLASTed against all our genomes to confirm their presence. PathogenFinder 2 was used to predict pathogenic capacity on humans from analyzed isolates (https://genepi.food.dtu.dk/pathogenfinder, last accessed February 2026 [53]. A custom *ad hoc* core‑genome multilocus sequence typing (cgMLST) scheme was generated using Ridom SeqSphere (Client Version 10.5.4, Server Version 10.5.0; Ridom GmbH) to assess the genetic relatedness of the 7 *S. pseudintermedius* isolates, that were whole- genome sequenced. The *S. pseudintermedius* strain NCTC5661 (Accession NZ_LR134267.1) was used as the seed genome for target definition. A total of 65 query genomes were included in the target discovery analysis. After automated locus identification and quality filtering, the ad hoc scheme comprised 1,079 cgMLST targets. Additionally, 1,223 loci were classified as accessory genome targets and 66 loci were discarded due to insufficient conservation or failing quality criteria.

The sequences generated in this study have been deposited in the NCBI Sequence Read Archive (SRA) and are available under BioProject accession number PRJNA1484583. Basic information about the genomic sequences is presented in S3 Table.

## Results

### Isolates

Overall, 46 (10.2%; 95% CI: 7.6–13.4) isolates belonging to SIG were detected in the 451 oral samples: 41 from *Z. californianus* (11.9; 95% CI: 8.7–15.8), including 38 *S. pseudintermedius* and three *S. delphini*; two from *M. angustirostris* (2.7%; CI: 0.3–9.3), including two *S. pseudintermedius;* two from *Callorhinus ursinus* (22.2%; CI: 2.8–60.0), including both*S. pseudintermedius* and *S. delphini*; and a single *S. pseudintermedius* isolate from *Phoca vitulina* (9.1%; CI: 0.2–41.3) (Table 1).

**Table 1:** Characteristics of the *Staphylococcus intermedius* group (SIG) isolates investigated, 2019-2022, Pacific coast of Southern California, USA.

Isolate	Host*	Species**	Phenotypic Antibiotic Resistance***	Antibiotic Resistance Genes	Exfoliative Toxin Genes	Leucocidin Genes	*spa* repeats****	MLVA code	MLVA type
818	Zc	Sp	PEN	*blaZ*, *blaI*, *blaR*	*siet*, *expB*	*lukS*, *lukF*	r01–r09–r02–r03–r03–r03–r29	0-5-6-5-0-1-16-1	Type 1
455, 943	Zc	Sp			*siet*, *expB*	*lukS*, *lukF*	n.t.	0-0-7-10-0-1-16-3	Type 10
939	Ma	Sp			*siet*, *expB*	*lukS*, *lukF*	n.t.	2-6-9-5-8-3-17-3	Type 11
296	Zc	Sp	PEN	*blaZ*, *blaI*, *blaR*	*siet*, *expB*	*lukS*, *lukF*	r01–r09–r02–r03–r03–r03–r06–r05	2-6-9-5-8-3-17-3	Type 11
300	Zc	Sp	TET	*tet*(M)	*siet*, *expB*	*lukS*, *lukF*	r01–r09–r02–r03–r03–r03–r06–r05	0-6-9-7-8-3-17-1	Type 12
302	Zc	Sd			*siet*, *expB*	*lukS*, *lukF*	r01–r09–r02–r03–r03–r03–r29	0-5-0-7-8-3-17-1	Type 13
309	Zc	Sp			*siet*, *expB*	*lukS*, *lukF*	r01–r09–r02–r03–r03–r03–r06–r05	0-6-7-2-8-3-17-1	Type 14
254	Zc	Sp			*siet*, *expB*	*lukS*, *lukF*	n.t.	0-6-7-2-8-3-17-1	Type 14
314	Zc	Sp			*siet*, *expB*	*lukS*, *lukF*	r01–r09–r02–r03–r03–r03–r29	2-5-9-5-8-3-17-3	Type 15
318	Zc	Sp			*siet*, *expB*	*lukS*, *lukF*	n.t.	0-6-7-7-8-3-17-1	Type 16
320	Zc	Sp			*siet*, *expB*	*lukS*, *lukF*	r01–r09–r02–r03–r03–r03–r06–r05	0-6-8-10-8-1-16-3	Type 17
328	Zc	Sp			*siet*, *expB*	*lukS*	r01–r09–r02–r03–r03–r03–r06–r05	0-6-8-10-8-1-16-3	Type 17
240	Zc	Sd			*siet*, *expB*	*lukS*, *lukF*	r01–r09–r02–r03–r03–r03–new2	0-6-7-7-8-3-17-0	Type 18
257, 280, 288	Zc	Sp			*siet*, *expB*	*lukS*, *lukF*	r01–r09–r02–r03–r03–r03–r29	0-5-9-7-0-3-17-1	Type 19
43	Zc	Sp			*siet*, *expB*	*lukS*, *lukF*	r01–r09–r02–r03–new	0-5-9-7-0-3-17-1	Type 19
312	Zc	Sp			*siet*, *expB*	*lukS*, *lukF*	r01–r09–r02–r03–r03–r03–r29	2-5-9-5-0-3-17-3	Type 2
16	Pv	Sp			*siet*, *expB*	*lukS*, *lukF*	r01–r09–r02–r03–r03–r03–r29	2-5-9-5-0-3-17-3	Type 2
266/1, 266/2	Zc	Sp	PEN	*blaZ*, *blaI*, *blaR*	*siet*, *expB*	*lukS*, *lukF*	r01–r09–r02–r03–r03–r03–r29	0-5-6-10-0-1-16-1	Type 20
24	Zc	Sp			*siet*, *expB*	*lukS*, *lukF*	r01–r09–r02–r03–r03–r03–r29	0-5-6-7-0-3-17-1	Type 21
30	Zc	Sp			*siet*, *expB*	*lukS*, *lukF*	r01–r09–r02–r03–r03–r03–r29	0-5-8-10-0-1-16-3	Type 22
28	Zc	Sp			*siet*, *expB*	*lukS*, *lukF*	n.t.	0-5-8-10-0-1-16-3	Type 22
55	Zc	Sp			*siet*, *expB*	*lukS*, *lukF*	n.t.	0-5-2-10-0-1-16-3	Type 23
34	Zc	Sp			*siet*, *expB*	*lukS*, *lukF*	n.t.	0-0-9-7-0-3-16-1	Type 24
32	Cu	Sd			*siet*, *expB*	*lukS*, *lukF*	n.t.	0-0-8-7-8-0-16-1	Type 25
734	Zc	Sp			*siet*, *expB*	*lukS*, *lukF*	n.t.	0-0-6-7-0-3-17-1	Type 26
770	Zc	Sp	PEN	*blaZ*, *blaI*, *blaR*	*siet*, *expB*	*lukS*, *lukF*	n.t.	0-0-6-10-0-1-16-1	Type 27
772	Zc	Sp			*siet*, *expB*	*lukS*, *lukF*	n.t.	0-0-9-7-0-3-17-1	Type 28
791, 793	Zc	Sp			*siet*, *expB*	*lukS*, *lukF*	n.t.	0-0-6-7-0-3-6-1	Type 29
808	Zc	Sp			*siet*, *expB*	*lukS*, *lukF*	r01–r09–r02–r03–r03–r03–new3	0-5-9-0-0-1-6-1	Type 3
834	Zc	Sp			*siet*, *expB*	*lukS*, *lukF*	r01–r09–r02–r03–r03–r03–r29	0-5-8-10-0-3-17-3	Type 4
1017	Zc	Sp			*siet*, *expB*	*lukS*, *lukF*	r01–r09–r02–r03–r03–r03–r29	0-5-9-0-0-3-17-1	Type 5
1021	Cu	Sp			*siet*, *expB*	*lukS*, *lukF*	r01–r09–r02–r03–r03–r03	0-5-6-7-0-3-6-1	Type 6
268, 272	Zc	Sp			*siet*, *expB*	*lukS*, *lukF*	r01–r09–r02–r03–r03–r03–r29	0-5-6-7-0-3-6-1	Type 6
885	Zc	Sp	PEN, TET	*blaZ*, *blaI*, *blaR*, *tet*(M)	*siet*, *expB*	*lukS*, *lukF*	n.t.	0-0-9-10-0-3-17-1	Type 7
883	Zc	Sd			*siet*, *expB*	*lukS*, *lukF*	n.t.	0-0-0-10-0-0-16-1	Type 8
956	Zc	Sp			*siet*, *expB*	*lukS*, *lukF*	t78r01–r09–r02–r03–r03–r03-new1	0-0-8-10-0-1-16-3	Type 9
749	Zc	Sp			*siet*, *expB*	*lukS*, *lukF*	n.t.	0-0-8-10-0-1-16-3	Type 9
764, 780, 778	Zc	Sp			*siet*, *expB*	*lukS*, *lukF*	n.t.	0-0-8-10-0-1-16-3	Type 9

*Host: Zc – *Zalophus californianus*, Ma – *Mirounga angustirostris*, Pv – *Phoca vitulina*, Cu – *Callorhinus ursinus*.

**Species: Sp – *Staphylococcus pseudintermedius*, Sd – *Staphylococcus delphini*.

***PEN – penicillin, TET – tetracycline.

****n.t: – non-typable, new1,2,3 – novel repeat.

### Antimicrobial susceptibility testing and detection of resistance genes

Thirty-nine isolates, including all *S. delphini*, were susceptible to all tested antimicrobial agents. Five isolates were only resistant to penicillin. One isolate displayed combined resistance to penicillin and tetracycline, and one isolate was solely resistant to tetracycline. DNA microarray analysis revealed that in all penicillin-resistant isolates, *blaZ* genes were detected, whereas resistance to tetracycline was mediated by the *tet*(M) gene (Table 1). Analyses of genomes via ResFinder and CARD confirmed that *blaZ* operon (*blaZ, blaI, blaR*) was present in penicillin resistant isolates, and *tet*(M) in tetracycline resistant isolate. None of the isolates analyzed were multidrug-resistant [54], and no methicillin-resistant *S. pseudintermedius* (MRSP) were observed. Among antibacterial biocide and metal-resistance genes, BacMet database, that is a bioinformatics resource that provides comprehensive information on antibacterial biocide and metal resistance genes, search revealed *arsB* (Arsenic pump membrane protein) and *fabI* (Enoyl-[acyl-carrier-protein] reductase [NADPH]) in all isolates. In addition, *arsC* was observed solely in *S. delphini*.However all had homology slightly higher than 80%.

### Genotyping of SIG-species and their virulence characteristic

Forty-two isolates shared the highest *rpoB* and *nuc* DNA sequence similarities with the type strain of *S. pseudintermedius* (*S. pseudintermedius* LMG22219^T^), whereas four isolates shared the highest *rpoB* and *nuc* DNA sequence similarities with the type strain of *S. delphini* (*S. delphini* NCTC12225^T^) (S1 and S2 Figs). In twenty-six isolates *spa* region could be amplified and sequenced and all yielded novel unknown *spa* types (Table 1). Twenty isolates were non-typable. A total of 29 MLVA types were identified (S3 Fig.), with their eight-digit profiles presented in Table 1. MLVA type 9 was the most prevalent (n = 5, 17.2%), followed by type 19 (n = 4, 13.8%) and type 6 (n = 3, 10.3%). Types 2, 10, 11, 14, 17, 20, 22, and 29 were each detected in two isolates (n = 2, 6.9%), while the remaining 18 types occurred as singletons (n = 1, 3.4%) (Table 1, S3 Fig.3).

**Fig 1:**
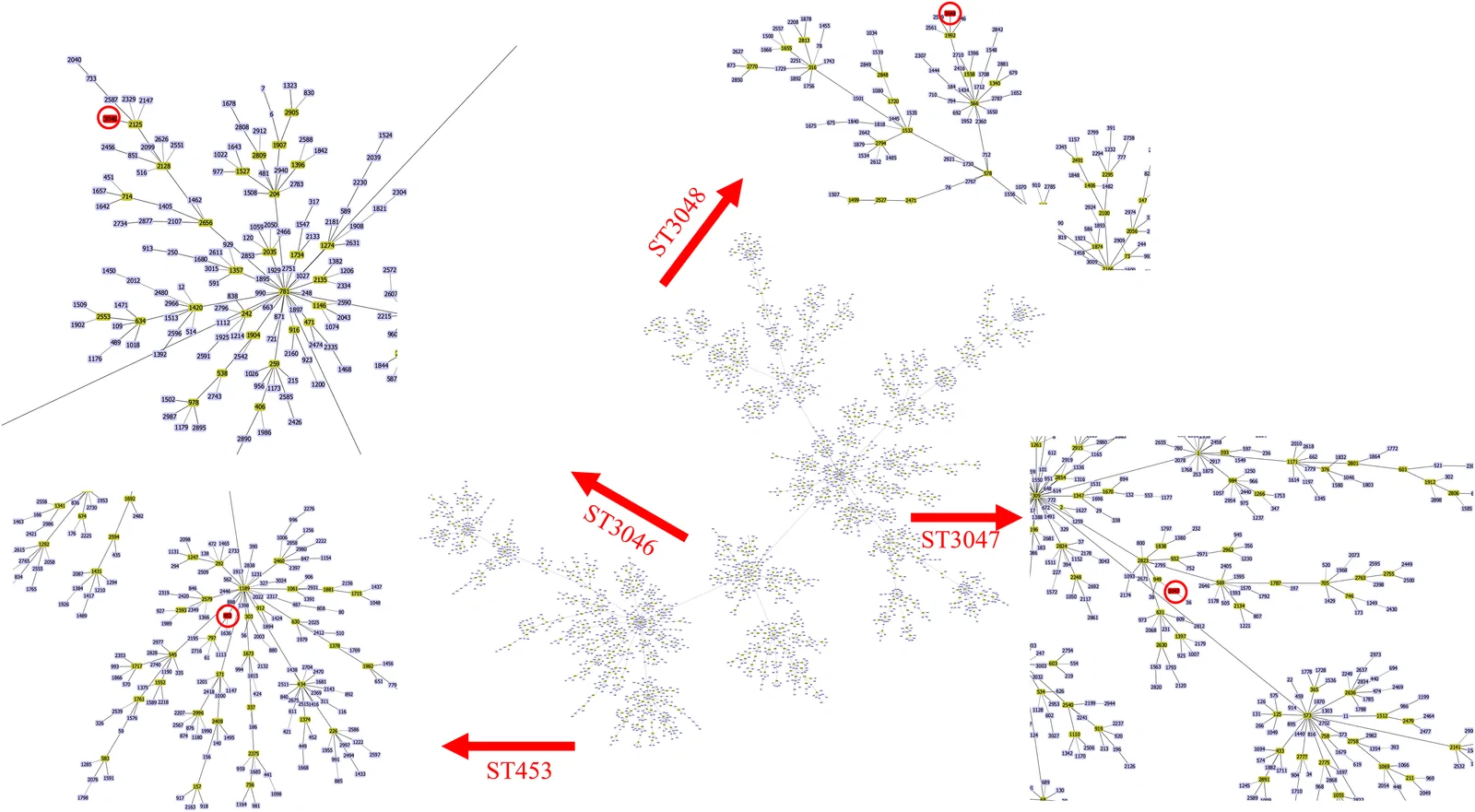
Population snapshot of *S. pseudintermedius* in the goeBURST analysis. A tree was constructed by connecting nodes at the Single-Locus Variant level to illustrate how STs were related.

**Fig 2:**
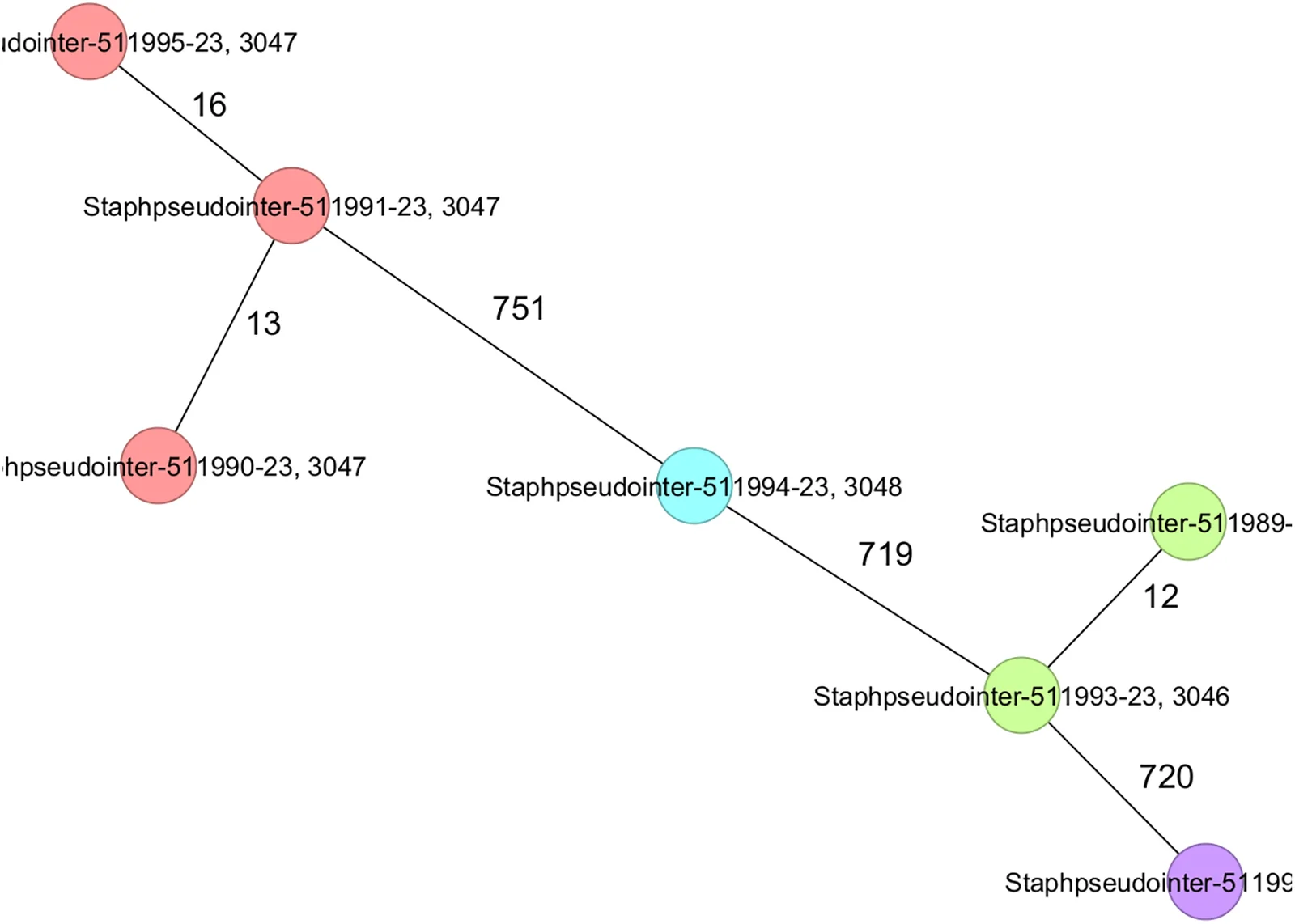
Minimum spanning tree for seven *S. pseudintermedius* isolates based on the cgMLST of *S. pseudintermedius.* Colors correspond to the sequence type.

Among eight selected isolates, wherefrom whole genomes were sequenced, seven were identified by JSpecies as *S. pseudintermedius* while one was *S. delphini*. Overall, four STs were observed among *S. pseudintermedius*, one isolate belonged to ST453 and three to novel STs (ST3046, ST3047, and 3048) (Fig 1). cgMLST also revealed similarities between isolates of the same STs (four allelic differences between ST3046 isolates and 9–11 allelic differences between ST3047 isolates) (Fig 2).

All isolates tested positive by PCR for the virulence-associated genes *siet*, *lukS*, and *expB*. The leukocidin subunit gene *lukF* was detected in all but one isolate (Table 1). *secCanine* or *expA* were not observed. Various virulence associate genes were detected, *ebpS, spsE, hlgA, hlgB, siet, lukS-I, lukF-I, lip, expB* were observed in all *S. pseudintermedius* isolates, whereas *icaA,* and *icaD* were detected in four isolates (Table 2). PathogenFinder 2 predicted that all isolates posed pathogenic potential on humans (all with mean prediction >0.8).

**Table 2:** Additional characterization of selected SIG isolates obtained from marine mammals via whole-genome sequencing.

Isolate/Alias*	MLST*	Virulence	Biocide and metal-resistance genes
818/511991-23	ST3047	*ebpS, spsE, hlgA, hlgB, lukS-I, lukF-I, lip*	*arsB, fabI*
296/511994-23	ST3048	*ebpS, spsE, hlgA, hlgB, lukS-I, lukF-I, lip, icaA, icaD*	*arsB, fabI*
266/2/511995-23	ST3047	*ebpS, spsE, hlgA, hlgB, lukS-I, lukF-I, lip*	*arsB, fabI*
32/511988-23	n.a.**	*spsE*	*arsB, arsC, fabI*
770/511990-23	ST3047	*ebpS, spsE, hlgA, hlgB, lukS-I, lukF-I, lip*	*arsB, fabI*
1021/511992-23	ST453	*ebpS, spsE, hlgA, hlgB, lukS-I, lukF-I, lip, icaA, icaD*	*arsB, fabI*
956/511993-23	ST3046	*ebpS, spsE, hlgA, hlgB, lukS-I, lukF-I, lip, icaA, icaD*	*arsB, fabI*
749/511989-23	ST3046	*ebpS, spsE, hlgA, hlgB, lukS-I, lukF-I, lip, icaA, icaD*	*arsB, fabI*

*Alias – internal identifier for each whole genome sequenced isolate.

**MLST – Multilocus sequence typing.

**n.a. – non-applicable.

## Discussion

The present study provides a detailed microbiological analysis of *S. pseudintermedius* and *S. delphini* isolated from the oral cavities of pinnipeds, assessing their antimicrobial susceptibility, genetic diversity, and pathogenic potential at the human-wildlife interface. While studies on SIG in animals, especially *S. pseudintermedius* in companion animals (dogs and cats), have been widely documented, information on infections, carriage, and role of these pathogens in marine mammals is scarce. Recent studies have advanced understanding of the pinniped intestinal microbiome and intestine-associated samples [55–57]. Knowledge of their skin- and mucosa-associated microbiota, particularly within the SIG, remains limited [55–57]. *S. pseudintermedius* has been previously isolated from the aural discharge in Atlantic harbor seals (*Phoca vitulina concolor*) from the Atlantic coast in the USA [58] and in the oral cavity of Weddell seals (*Leptonychotes weddellii*) on James Ross Island and Seymour Island, Antarctica [59]. *S. delphini* has been previously isolated from purulent skin lesions of captive dolphins in Italy, although the specific host species was not identified in the original manuscript [7]. The presence of *blaZ* and *tet*(M) resistance genes is a common observation in *S. pseudintermedius* [60,61].

In line with previous studies, *spa* typing of *S. pseudintermedius* was an effective tool for specific isolates but lacked universal applicability across all lineages [62]. Furthermore, the MLVA scheme developed during the present study demonstrated superior discriminatory power. Its cost-effectiveness and technical simplicity make it a valuable first and/or additional step in the polyphasic molecular characterization of *S. pseudintermedius* and/or *S. delphini* consistent with previous studies applying it on other bacteria [63]. Additionally, MLVA serves as an ideal genotyping tool in laboratories with limited genomic infrastructure. Very recently, another MLVA scheme for *S. pseudintermedius* was developed [64] and demonstrated to be well suited for typing *S. pseudintermedius*. In contrast to our study, Afema et al. [64] utilized capillary electrophoresis, which enables high throughput in laboratories equipped with this technology. Both MLVA schemes could potentially be merged for use with classical electrophoresis if they meet the necessary criteria, and immediately for capillary electrophoresis in future studies to achieve even greater discriminatory power.

A direct comparison of the isolates from the previously mentioned reports with those obtained in our study presents several methodological challenges, namely, remote geographical origin and different host species. In the *S. pseudintermedius* PubMLST isolate database, there is a single entry associated with the marine mammals. This strain was isolated from the oral cavity of a healthy Weddell seal (*Leptonychotes weddellii*) in Antarctica. This particular strain belonged, to ST723 which is only associated with this animal species, and they are only distantly related (Triple Locus Variants (TLV)) STs in PubMLST database (*S. pseudintermedius* PubMLST, https://pubmlst.org/organisms/staphylococcus-pseudintermedius, last accesses February 2026.). Among four STs obtained during the present study, ST453 has already been established and were associated with canine urinary tract infections from Germany. ST453 had 13 Single Locus Variants (SLV). Three other novel STs ST3046, ST3047, and ST3048 have one SLV (ST2125, originated from canine MRSP in Brazil), no SLV, and one SLV (ST1992, from a healthy dog from Texas, USA), respectively (*S. pseudintermedius* PubMLST, https://pubmlst.org/organisms/staphylococcus-pseudintermedius, last accesses February 2026). All of these STs including loci combination of *S. pseudintermedius* P8688 (4-12-1-1-102-1-1 (novel ST still not generated, [59]) are distinct lineages and none are from widely recognized, large-scale lineages often associated with various infections in terrestrial animals like clonal complex (CC) 71, CC45 or CC68 [60].

The nearly ubiquitous presence of exfoliative toxin genes (*siet*, *expB*) and the leucocidin locus *lukI*, is in concordance with previous reports [29,62]. Furthermore, WGS revealed additional virulence-associated genes *ebpS* (elastin-binding protein), *spsE* (fibrinogen-binding protein), *hlgA* and *hlgB* (gamma-hemolyins), and *lip* (lipase). In some isolates, biofilm regulation genes *icaA* and *icaB* were observed, further aligning with the previous studies [59,60].

Our study, while providing unique insights into the presence of SIG within marine mammal populations, has some limitations. First, the study relied exclusively on samples collected from stranded animals. This cohort may not be representative, potentially leading to sampling bias. The geographical scope of this study is restricted to coastal Los Angeles County, which limits the representativeness of the findings. Based on our data, we cannot conclusively determine whether SIG in marine mammals represents transient colonization or is part of the normal microflora of these animals. However, the identification of very similar isolates belonging to the same ST across different individuals, locations, and temporal scales suggests that SIG may indeed constitute a component of the natural marine mammal microbiome. Finally, the potential for environmental or terrestrial contribution cannot be dismissed, because most of the Los Angeles metropolitan area drains into Santa Monica Bay. The influence of terrestrial runoff and sewage effluent on the microbial profiles observed in these marine hosts remains a critical variable for future investigations.

The detection of zoonotic SIG species (*S. pseudintermedius* and *S. delphini*) in stranded marine mammals raises important considerations for public health and wildlife management. While *S. pseudintermedius* is recognized as an emerging zoonotic pathogen capable of causing opportunistic infections in humans [65,66], the zoonotic potential of *S. delphini* has only recently been documented [67]. The presence of these bacteria in the oral cavities of marine mammals frequenting beaches and rehabilitation facilities along the highly urbanized Southern California coastline creates potential interfaces for human exposure. However, the absence of multidrug resistance and methicillin resistance in our isolates suggests that, at present, these marine-associated lineages pose a low clinical threat. Nevertheless, the potential for horizontal gene transfer in coastal environments, where terrestrial runoff, wastewater discharge, and marine ecosystems converge, could facilitate the acquisition of resistance determinants by these marine-adapted lineages. The distinct phylogenetic positioning of our novel STs (ST3046, ST3047, ST3048) and their separation from major terrestrial clonal complexes (CC71, CC45, CC68, CC551, CC556, CC1431, CC363 and CC1631 [68] may reflect ecological barriers to gene flow between marine and terrestrial SIG populations. Understanding whether marine mammals serve as transient carriers, environmental accumulators, or true reservoirs for zoonotic SIG lineages is essential for informed risk assessment.

## Conclusion

This investigation reveals that marine mammals along the Southern California coast harbor distinct and diverse lineages of *S. pseudintermedius* and *S. delphini* (both zoonotic), largely separate from the common terrestrial clones associated with domestic animals and humans. The identification of novel SIG lineages in these marine species underscores the unexplored microbial diversity present in oceanic ecosystems and challenges our current understanding of staphylococcal ecology and host adaptation. By leveraging stranded marine mammals as sentinels of ocean health, this study identifies novel SIG lineages in these marine species, underscoring the unexplored microbial diversity present in oceanic ecosystems, thereby challenging our current understanding of staphylococcal ecology and host adaptation. It also establishes a critical baseline for One Health surveillance at the interface of marine wildlife, environmental, and human health.

## Supporting information

S1 Table Full animal data. (XLSX)

S2 Table Primers and amplification conditions of the VNTR loci. Locus name was determined by the gene position in reference genome ED99 and “sp” standing for *Staphylococcus pseudintermedius*.
(XLSX)

S3 Table Basic information about the genomic sequences. (XLSX)

S1 Fig Neighbor-Joining method phylogenetic tree generated of *rpoB* sequences including type strains of SIG. Bootstrap values (expressed as percentages of 100 replications) greater than 75% are shown at branching points. The evolutionary distances were computed using the Maximum Composite Likelihood method.
(TIF)

S2 Fig Neighbor-Joining method phylogenetic tree generated of *nuc* sequences including type strains of SIG. Bootstrap values (expressed as percentages of 100 replications) greater than 75% are shown at branching points. The evolutionary distances were computed using the Maximum Composite Likelihood method.
(TIF)

S3 Fig Clustering analysis of MLVA of isolates tested using the categorical coefficient (also called Hamming’s distance) and the unweighted pair group method with arithmetic mean clustering method (UPGMA). Isolates sharing the same MLVA type are separated by a “/”.
(JPG)
